# Capillary and venous lactate measurements with a handheld device compared to venous blood-gas analysis for emergency patients

**DOI:** 10.1186/s13049-018-0510-5

**Published:** 2018-06-05

**Authors:** David Stoll, Erling Englund, Helene Hillborg, Stefan Vedin, Agneta Larsson

**Affiliations:** 1 Ambulance Services, Sundsvall Hospital, Västernorrland County Council, 851 86 Sundsvall, Sweden; 2 Research and Development, Sundsvall Hospital, Västernorrland County Council, Sundsvall, Sweden; 3 Karolinska Institutet, Department of Physiology and Pharmacology, Stockholm, Sweden

**Keywords:** Handheld lactate analyzer, Lactate, Prehospital emergency care, Point of care (POC)

## Abstract

**Background/aim:**

Early identification of lactate levels may have a large impact on triage classification and assist in identifying critically ill patients. A handheld device provides a rapid and timesaving measurement of lactate levels adapted to work in a prehospital care setting. I.e., the device is small, fast, and easy-to-use. The aim of this study was to evaluate the Accutrend Plus handheld lactate analyzer in comparison to the reference in-hospital method.

**Methods:**

Patients triaged as minimum yellow according to the RETTS System (Rapid Emergency Triage and Treatment System) and transported to hospital by ambulance were selected and a written consent to participate was obtained prior to inclusion in the study. Capillary (CAP) and venous (VEN) blood were analyzed with Accutrend Plus (AP). Venous blood samples were analyzed at the local hospital laboratory (GEM premier 4000) within 20 min from sampling. All sampling was conducted by two registered nurses specially trained in prehospital care.

**Results:**

480 lactate measurements were performed in 160 patients. The mean difference between measurements in capillary blood compared with the reference method was 0.7 mmol/L and for venous blood 0.9 mmol/L. The limits of agreement from the Bland-Altman plot was − 0.9 to + 2.5 mmol/L and and − 0.1 to + 1.9 mmol/L, for CAP and VEN compared with GEM.

**Conclusion:**

Our results shows low accuracy and low precision with VEN / CAP measurements of lactate compared to reference GEM.

## Background

In prehospital care, it is important to triage patients correctly based on the severity of their injuries/conditions [1]. Measurement of lactate levels in addition to standard parameters, e.g. vital signs, might improve the assessment of severity, choice of level of care and destination for the patient. However, the significance of lactate levels cannot be determined accurately until an adapted equipment is available in a prehospital environment.

The most common triage system used prehospitally and in emergency wards in Sweden, is at present RETTS (Rapid Emergency Triage and Treatment System), which is high-sensitive in detecting critical patients [2]. The RETTS system uses vital signs, e.g. respiratory rate, oxygen saturation, pulse and blood pressure combined with signs and symptoms of the patient. The system also considers medical history and experiences on development of different medical conditions. The vital signs used by the RETTS system (see above) are not sufficient to reflect anaerobic metabolism and acidosis which is of great interest in the valuation of critical conditions. Lactate levels are often considered to be better resuscitation endpoints than standard vital signs. Early identifications of lactate levels may have a large impact on triage classification and assist in identifying critically ill patients [3, 4]. An ideal prehospital device would provide a rapid analyze and be adapted to a prehospital setting i.e., carry little weight and be of small size as well as being easy to handle at bedside and have simple hygienic procedures [4].

Previous studies Jansen et al. [5] examined the association between elevated lactate detected prehospital and mortality rate. Baldari et al. [6] and Pyne et al. [7] investigated the reliability of the AP in young healthy athletes where the results showed a linear relationship to the reference method taken from capillary blood samples. Pattharanitima et al. [8] demonstrated a high correlation and good agreement between arterial and capillary lactate values, as well as high correlations between arterial and central venous lactate values of 30 sepsis patients with AP in hospital settings. However, we have not found any studies that have examined the reliability of Accutrend Plus (AP) using capillary and venous blood samples in prehospital environment.

Arterial lactate analysis is the conventional method used to analyze lactate. However, arterial blood samples require technologies and equipment’s that are not suitable for prehospital settings. The aim of this study was to test the Accutrend Plus handheld lactate analyzer to evaluate its performance in a prehospital environment in comparison to the reference in-hospital method.

## Methods

A prospective observational study was conducted in the Ambulance Services in Västernorrland County Hospital Sundsvall, in association with Karolinska Institutet, from April 1st, 2014 to April 1st, 2015.

### Subjects

**Fig. 1:**
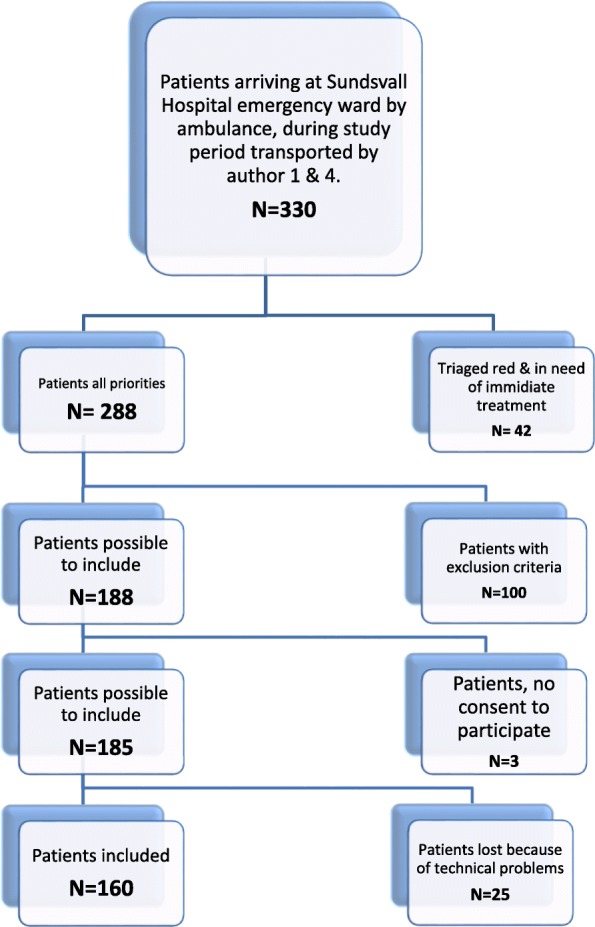
Flowchart

One hundred sixty patients transported to hospital by ambulance were included in the study (Fig. 1). Inclusion criteria were patients older than 18 years of age who, according to the triage system *Rapid Emergency Triage and Treatment System* (RETTS), were triaged red, orange or yellow by two ambulance nurses (1st and 4th authors). Red priority is defined as patient in need of medical assessment immediately, i.e. to see a doctor instantly on arrival to the emergency ward. Orange priority is defined as a patient in need of medical assessment within 20 min and yellow priority within 120 min [2] after arrival to the emergency ward.

Patients in palliative treatment, circulatory arrest, with a history of coagulopathy, or requiring immediate medical treatment for life threatening conditions, and those patients who could not understand or receive information about the study were excluded.

### Ethics

The study was approved by the regional committee for medical and health research ethics at Umeå (Ref. 2014–14-31 M). Each participant was given a verbal and written information of the study. All participation was voluntarily and a written consent to participate was obtained prior to inclusion.

### Procedure

Data were collected by two specially selected ambulance nurses (1st and 4th authors) with more than 10 years’ experience of blood sampling. Only patients presented to the ambulance service when the two nurses were on duty could be included. Patients transported to hospital received peripheral venous access in the upper limb before leaving the ambulance, according to local medical guidelines. Capillary and venous blood samples were collected simultaneously as venous access within a 5–10 min window from arrival. Venous sampling was conducted without a tourniquet. All samplings were done simultaneously. No saline or drugs were used in the cannula before samples were taken. Blood was not aspirated and discarded before sampling. The analysis of the AP took approximately 2–3 min. The analysis of the lactate sample in the hospital’s local laboratory was conducted within 20 min after sampling. This measure was used as reference (GEM Premier 4000).

Measurement of lactate was analyzed as follows:

- Capillary (CAP) - a drop of blood from the fingertip analyzed by AP.
- Venous (VEN) - venous blood collected by back-flow from the venous access and analyzed by AP.
- Venous Intrashop Paired analyzed by hospital - four milliliter venous blood was collected by back-flow from the pre-existing cannula into a lithium heparinized standard sample tube. The sample was analyzed at the local hospital laboratory by GEM Premier 4000 (GEM). GEM sample was used as the reference, and analyzed within 20 min after collection of the sample.

All 160 patients had their lactate measured three times (CAP, VEN and GEM) giving a total of 480 samples. AP and GEM samples were calibrated regularly according to instructions by the manufacturers.

### Lactate analyzers

The AP lactate analyser (Roche, Diagnostic international Ltd.) is a portable, battery-driven device that weighs approximately 140 g. AP measures whole blood lactate values sampled from capillary blood. The measuring range is 0.8–22 mM. A drop of blood is applied on a chemistry strip and analyzed with a reflectance photometric method. The result is displayed with a turnaround time of 60 s.

GEM Premier 4000 (Instrumentation Laboratory) is a compact (20 kg) system used for whole blood analyzes and designed for emergency- and central laboratory testing. The analyzer measures blood gases of a single whole blood sample with an amperometric and potentiometric method.

### Statistics

One hundred sixty patients were estimated to give a sufficient sample size to be able to detect an ICC (intraclass correlation coefficient) of 0.8 when intraclass- correlation in the null hypothesis is 0.7. This is based on an F-test and on two observations per individual with a power of 90% and a significance level of 5% [9].

The agreement of AP compared with the reference method was determined using Bland Altman plots. This was used to visualize both the accuracy (bias), and precision. The sample size was also in line with recommendation for Bland-Altman plot [10]. Correlation between AP and the reference method GEM was estimated by intraclass correlation coefficient (ICC (2.1)), where ICC values greater than 0.8 was interpreted to be in absolute agreement. Two cut-points, 2 and 3.5, were used to describe how the capillary and venous lactate measurement behave for different severity intervals [5]. Parametric methods were used as the size of sample permits the assumption normal distribution. Statistical analyzes were performed using IBM SPSS 23. Graphics was created in Origin 9.0.

## Results

Four hundred eighty lactate measurements were analyzed from 160 patients. Mean age was 70.1 years, and 50% were women (Tables 1 and 2).

**Table 1:** Patients characteristics (*n* = 160)

Variables\Triage category	Yellow	Orange	Red
	*n* = 65	*n* = 81	*n* = 14
Age			
Mean (Range)	70.7 (40–96)	70.2 (20–96)	67.8 (45–83)
Gender			
Female	42 (65%)	35 (43%)	3 (21%)
Accutrend Plus			
Capillary ≤3.5 mmol/L	54 (83%)	71 (88%)	8 (57%)
Venous ≤3.5 mmol/L	60 (92%)	74 (91%)	6 (43%)
Gem Premier 4000			
Venous ≤3.5 mmol/L	63 (97%)	79 (98%)	9 (64%)

**Table 2:** Lactate levels measured with Accutrend Plus and Gem 4000 (*n* = 160)

Analyser	≤2 mmol/L	> 2 - ≤3.5 mmol/L	> 3.5 mmol/L
	Number	Number	Number
Accutrend Plus			
Capillary	68	65	27
Venous	48	92	20
Gem Premier 4000			
Venous	128	23	9

The analysis with AP measured an ICC level of 0.76 (CI: 0.27–0.90) in CAP and 0.79 (CI: 0.00–0.94) in VEN. The mean difference (Bias value) of all measurements in CAP compared with GEM was 0.7 mmol/L. The level of agreement indicated that for 95% of the measurements differences would be between − 0.9 to + 2.5 mmol/L.

**Fig. 2:**
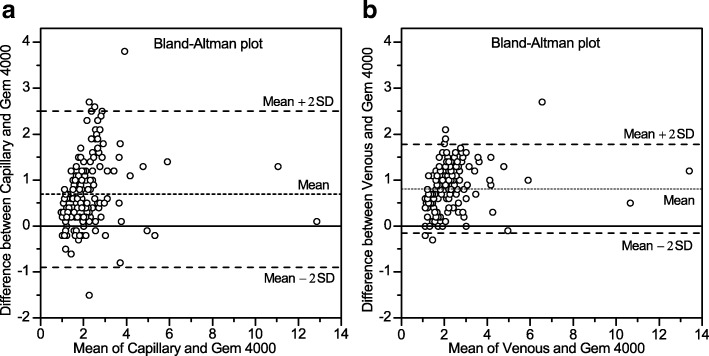
**a, b** The Bland-Altman plots describes the level of agreement between the CAP/VEN measurements and the reference method. The Y-axis represents the differences between CAP/VEN and GEM. The X axis represents the mean of CAP/VEN and GEM. The dotted lines displays mean of the differences and the 95% limits of agreements

The mean difference (Bias value) of all measurements in VEN compared with GEM was 0.9 mmol/L. The level of agreement indicated that for 95% of the measurements differences would be between − 0.1 to + 1.9 mmol/L (Fig. 2a and b.

## Discussion

We have in our study added data on capillary and venous sampling with a device that seems practical to use prehospitally. Our study of CAP and VEN sample analysis showed generally higher values (mean bias, 0.7 and 0.9 and upper level of limits of agreement, 2.4 and 1.9 mmol/L) than the reference method GEM (Fig. 2). This agrees with results from Baldari et al. [6] that also demonstrated higher levels of lactate in healthy athletes when using handheld analyzers. The Bland-Altman plot shows high bias in both VEN and CAP, although somewhat higher in VEN. The precision is superior with VEN than CAP which may be more important than the bias. High precision is associated with low risk for underestimation, which is, from a clinical point of view, safer. The Bland-Altman plot also illustrates if the variation of differences is constant through different values on the x-axis. It seems to be the case for values under 4, but it’s difficult to assess if the same is true for values > 4 as only a few values are > 4 in our study [10]. Moreover, no sign of systematic change in bias in the Bland-Altman plot. In Table 3 only small changes in mean bias was found when GEM values were 0–2 mmol/L and 2–3.5 mmol/L (Fig. 2). Our results show a low accuracy with CAP, as well as with VEN analyzed with the handheld AP compared to the reference method GEM. This is in discrepancy to Baldari [6] and Pyne et al. [7] who investigated the reliability of the AP in young healthy athletes and where the results showed a good linear relationship with the reference method using capillary blood samples. Pattharanitima et al. [8] also showed a high correlation between arterial and capillary lactate values, as well as high correlation between arterial and central venous lactate values in 30 septic inhospital patients using AP. Our results concur with Datta et al. [11] who demonstrated a poor agreement of capillary lactate analyzes in comparison to peripheral venous lactate measurements.

**Table 3:** Mean, standard deviation and bias of Accutrend Plus and GEM

	GEM 0 - ≤2			GEM > 2 - ≤3.5			Total		
	Mean	SD	Bias	Mean	SD	Bias	Mean	SD	Bias
Accutrend Plus									
Capillary	2.18	0.86	0.77	3.29	0.93	0.47	2.55	1.51	0.72
Venous	2.27	0.60	0.86	3.70	0.74	0.88	2.71	1.48	0.88
GEM									
Venous	1.41	0.34	–	2.82	0.72	–	1.83	1.37	–

Data are presented as mean, standard deviation (SD) and bias for values were GEM is between 0 and 2, 2–3.5 as well as Total. Bias = mean difference between AP lactate levels and GEM

The limits of agreement show an existing bias in both lactate measurements but slightly larger for VEN. The limit is wider for CAP compared to VEN. The scatter of the differences is constant in increasing values on the x-axis, both for CAP and VEN-values. The bias in CAP is lower than in VEN, but for values below 2 mmol/L and between 2 and 2.5 mmol/L bias is more stable for VEN (Table 3).

### Limitations

Time between sample and analysis was seconds for the handheld analyzer. The blood samples were collected upon arrival by two nurses (authors 1 and 4) using the same procedure for all patients included. No local injuries or issues in the limbs where the samples were collected where found for any of the patients included in the study during the stay at the emergency ward. All samples tested with GEM were analyzed within 20 min (30 min stipulated by manufacturer). We consider our bias concerning differences in sampling procedures minute.

As shown in Table 1 the majority (91,2%) of the included patients were triaged orange/yellow and only a few red patients could be included. This may have selected a category of patients with similar lactate levels and minimized the spread of our measurements. The discrepancy to the results found by Baldari [6] and Pyne et al. [7] who investigated capillary samples in young healthy athletes and Pattharanitima et al. [8] who analyzed capillary samples from septic patients could perhaps be explained by the difference in the selection of patients. Where our study had a relatively large number of patients (*n* = 160 triaged mainly as orange/yellow priority) with a similar level of lactate the Baldari study [6] showed a wider range of lactate levels. The red/orange patients included with possible low blood-pressure may have had peripheral vasoconstriction. This may account for differences between VEN and CAP levels.

We found that lactate levels measured with CAP, as well as VEN, were higher than the values measured with GEM (Fig. 2). The spread of 2 mmol/L above values analyzed by GEM was more frequent for CAP than for VEN. Baldari et al. [6] found that the deviation with the handheld compared to stationary blood-gas analyzers escalated with higher lactate values. The measured levels of lactate in our study were generally lower than 6 mmol/L which gives a small spread of the results and may account for our small deviation from reference. Further studies with patients in critical conditions with a wider spread of lactate levels are needed to be able to fully evaluate the potential use of CAP samples.

## Conclusion

Our results demonstrate low accuracy and relatively low precision with VEN / CAP measurements of lactate compared to reference GEM. Our results also suggest that VEN are safer than CAP measurements because of its better precision.

## Abbreviations

AP: Accutrend plus
CAP: Capillary
GEM: GEM premier 4000
VEN: Venous
